# Endophytic actinobacteria from wild medicinal plants are a natural source of insecticide to control the African cotton leafworm (*Spodoptera littoralis*)

**DOI:** 10.1186/s13568-023-01550-x

**Published:** 2023-05-15

**Authors:** Mohamed K. Diab, Hala M. Mead, Mohamad A. Khedr, Mohamed S. Nafie, Abdelghafar M. Abu-Elsaoud, Amro Hanora, Sahar A. El-Shatoury

**Affiliations:** 1 Agricultural Research Center, Pest Physiology Department, Plant Protection Research Institute, Giza, 12311 Egypt; 2 Agricultural Research Center, Cotton Leafworm Department, Plant Protection Research Institute, Giza, 12311 Egypt; 3 Faculty of Science, Chemistry Department, Suez Canal University, Ismailia, 41522 Egypt; 4 Faculty of Science, Botany & Microbiology Department, Suez Canal University, Ismailia, 41522 Egypt; 5 Faculty of Pharmacy, Microbiology Department, Suez Canal University, Ismailia, 41522 Egypt

**Keywords:** Cyromazine, Endophytes, Medicinal plants, *Spodoptera littoralis*, *Streptomyces* sp., 4-nitrophenol

## Abstract

Insecticide resistance in agricultural pests has prompted the need to discover novel compounds with new modes of action. We investigated the potency of secondary metabolites from seventy endophytic actinobacteria against laboratory and field strains of *Spodoptera littoralis* (fourth instar), comparable to the bioinsecticide spinetoram (Radiant SC 12%). Endophytes from *Artemisia herba-alba* and *A. judaica* were highly effective. Chemical profiling of the most potent metabolite of the strain *Streptomyces* sp. ES2 was investigated using LC-QTOF-MS-MS technique, and the activity was validated through molecular docking studies. Metabolic extracts from actinobacteria belonging to *Streptomyces*, *Nocardioides*, and *Pseudonocardia* showed immediate and latent death to the *Spodoptera littoralis* fourth instar larvae. The metabolite from strain ES2 has shown the most promising and significant histopathological and inhibitory effects on the fourth instar larvae. ES2 metabolite caused lesions in the body wall cuticle, indicating a different mode of action than that of Radiant. Chemical profiling of ES2 showed the presence of cyromazine (molt inhibitor), 4-nitrophenol, and diazinon as key constituents. In conclusion, these findings suggest that secondary metabolites from endophytic actinobacteria inhabiting wild medicinal plants can be a sustainable source for promising natural biocontrol agents. This is the first illustration of the insecticidal activity of *Artemisia* spp. microbiome, and natural cyromazine synthesis by actinobacteria.

**Supplementary Information:**

The online version contains supplementary material available at 10.1186/s13568-023-01550-x.

## Introduction

The cotton leafworm, *Spodoptera littoralis* (Boisduval 1833) (Lepidoptera: Noctuidae) is commonly distributed worldwide. It is one of the most destructive agricultural pests within the subtropical and tropical range (Pasiecznik et al. 2005). *S. littoralis* causes considerable annual damage to plants belonging to 44 different families, including cotton, ornamentals, vegetables, and economically important crops. The control of *Spodoptera* spp. requires the massive use of insecticides. These insects have acquired resistance to all chemical families, including organophosphates, carbamates, and pyrethroids, as well as a more recent family, diamides (Hilliou et al. 2021). One of the most effective insecticides is cyromazine, a triazine derivative. Cyromazine's physiological effects on larvae include abnormal melanization and sclerotization of the cuticle, necrotic lesions, insect body rupture, and death. (Pener and Dhadialla 2012). Many biochemical and genetic investigations on the mode of action of pesticides in *Spodoptera* spp. have been conducted (Fahmy and Dahi 2009; Rehan and Freed 2014; Bird and Drynan 2023). However, the mechanistic basis of cyromazine toxicity in insect has only been recently investigated (Chang et al. 2022). Currently, new technologies based on microbe- and plant-derived insecticides have become essential because *S. littoralis* has acquired resistance to the traditionally used pesticides (Hazaa et al. 2020).

Actinobacteria are Gram-positive bacteria with high G + C DNA content that constitute one of the largest bacterial phyla. They are of great biotechnological applications, as producers of a plethora of bioactive secondary metabolites with wide industrial, medical, and agricultural applications (Barka et al 2016). Actinobacteria have been reported as endophytes within live tissues of various plant species. Endophytic actinobacteria are an important source of bioactive metabolites, including those for agricultural applications (El-Tarabily and Sivasithamparam 2006). The most frequently observed species belonged to the genera *Streptomyces* and *Micromonospora* (El-Tarabily 2019; Toghueo and Boyom 2019; El-Tarabily 2021). Endophytic actinobacteria are particularly important, if not essential, for plant growth and development. They have been shown to protect plants against different soil-borne plant pathogens including *Fusarium Pythium spp.* (El-Tarabily 2009; Alblooshi 2022).

Previous studies have shown high insecticidal activities of essential oils from two wild plants, *Artemisia herba-alba* and *Artemisia Judaica*, against coleopteran pests, such as *Orysaephilus surinamensis* (Bachrouch et al. 2015) and *Tribolium casteneum* (Deb 2020). Therefore, a promising strategy to discover bioactive products is to investigate endophytic microbes, particularly those inhabiting wild and medicinal plants. This is due to the abilities of endophytes to utilize unique constituents of the medicinal plant to produces bioactive metabolites with distinct and novel structures (Tanvir et al. 2019). *Streptomyces albus*, is an example of endophytes from the drunken horse grass, *Achnatherum inebrians* which exhibited more than 90% mortality in the cotton aphid, *Aphis gossypii* Glover (Shi et al. 2013). *Streptomyces* sp. from *Stemona sessilifolia* (a traditional Chinese medicinal plant) produced ten new endostemonines compounds, with strong lethal activity against *Aphis gossypii* (Zhao et al. 2020). It's important to note that the development of self-protecting plants that are enhanced with the powerful endophytes in their microbiomes is another important aspect of the future of endophyte-derived pesticides. The use of plant microbiome augmentation may allow agriculturists to more sustainably produce crops at a reduced cost (White et al. 2019).

However, only few microbial metabolites have reached the market for pest control (Kvakkestad et al. 2020). Those included, avermectins which are produced by *Streptomyces avermitilis*, isolated from soil. It inhibits the neurotransmission in the insect due to an increased flow of chloride ions into the cell (Siddique et al. 2014). Spinosyns are a distinct family of natural product-based insecticides that currently include two insecticidal active ingredients, spinosad, a naturally occurring spinosyn combination, and spinetoram, a semi-synthetic spinosyn product (Sparks et al. 2021; Bird and Drynan 2023). Spinosad (a spinosyns mixture, produced by the soil actinobacterium *Saccharopolyspora spinosa*) acts on nicotinic acetylcholine receptors, causing death due to disruption of the nervous system (Kim et al. 2010). In addition, 30 butenyl-spinosyns of higher potency are produced by *Saccharopolyspora pogona* (Rang et al. 2020). However, due to the extensive and extended use of pesticides, *S. littoralis* develops a tolerance, necessitating the constant search of alternatives to manage the pest.

This study aimed to investigate the insecticidal potential of the secondary metabolites produced by endophytic actinobacteria isolated from six medicinal plant species in the World Heritage Site of Saint Catherine (WHS No. 954), South Sinai, Egypt. Up to our knowledge, no previous studies exist on insecticides derived from the endophytes of this conservative area. This site can represent a source of unique and diverse endophytes from its endemic plants. The most potent insecticidal activities were assessed on the fourth instar larvae of *Spodoptera littoralis* field strain, compared to the laboratory strain. The toxicological, histopathological, and biochemical effects of the most potent actinobacterial metabolites were evaluated, as an initial effort to introduce a natural control agent for cotton leafworm.

## Materials and methods

All chemicals and solvents were purchased from Sigma Aldrich (Chemie GmbH, Taufkirchen, Germany). The commercial pesticide Radiant SC 12% (Dow Agrosciences, Canada; CAS Number: 187166-40-1) was obtained from the Plant Protection Research Institute, Ministry of Agriculture, Egypt. Radiant’s active ingredient is spinetoram, a second generation of the spinosyns.

### Endophytic actinobacteria and culture conditions

Seventy actinobacteria strains, previously isolated from six wild medicinal plant species (F. Compositae) in South Sinai, Egypt, were studied (El-Shatoury et al. 2013). The source-plant of the strains and their generic identity, based on the standard chemotaxonomy analysis is illustrated in Table 1. The strains were preserved as spore suspensions in 20% v/v glycerol (El Nasr Pharmaceutical Chemicals Co., ADWIC, Egypt) at − 15 °C, and were refreshed on starch casein agar (Sigma-Aldrich, Chemie GmbH, Taufkirchen, Germany), as described by Kieser et al. (2000).

**Table 1:** Numbers and generic identification of the actinobacteria isolated from six wild medicinal plant species (F. Compositae) in South Sinai, Egypt

Genus of Actinobacteria	Host plant, “common name” and Latin name					
	“White wormwood” *Artemisia herba-alba* Asso	“Chicory” *Scariola orientalis* (Boiss.)	“Sinai tansy” *Tanacetum sinaicum* (Fresen.)	“Judean Wormwood” *Artemisia judaica* L	“Lavender Cotton” *Achillea fragrantissima* (Forssk.)	"Globe Thistle” *Echinops spinosus* L
*Streptomyces* spp.	21 strains, 30%	5 strains, 7.1%	2 strains, 2.9%	5 strains, 7.1%	2 strains, 2.9%	2 strains, 2.9%
*Nocardiopsis* spp.	5 strains, 7.1%		1 strain, 1.4%	1 strain, 1.4%		
*Nocardioides* spp.	16 strains, 22.9%	1 strain, 1.4%		1 strain, 1.4%	1 strain, 1.4%	
*Pseudonocardia* sp.	1 strain, 1.4%					
*Nocardia* sp.	1 strain, 1.4%					
*Kibdellosporangium* sp.					1 strain, 1.4%	
*Promicromonospora* sp.				1 strain, 1.4%		
Unknown spp.	2 strains, 2.9%	1 strain, 1.4%				
Total %	65.7%	9.9%	4.3%	11.3%	5.7%	2.9%

(%) indicates percentage of genus recovery from each plant

### *Spodoptera littoralis* larvae rearing

The laboratory *Spodoptera littoralis* larvae (L-larvae) was reared under constant laboratory conditions, at the Plant Protection Research Institute, Agricultural Research Center, Zagazig, Egypt. It was reared in an incubator at a temperature of 26 ± 2 °C, a relative humidity of 65 ± 10% RH, and 16: 8 h of light and dark, respectively, according to Mansour et al. (1966). The L-larvae were reared for thirty successive generations to guarantee that it is free from any resistance to pesticides. The field *Spodoptera littoralis* larvae (F-larvae) were collected from the local open field at Sharqia Governorate, Egypt (30°37'O6.6"N 31°32′54.8"E). They were transferred to the laboratory and reared for two successive generations, as described above.

Both L- and F- larval instars were fed on fresh castor bean leaves, *Ricinus communis* L., until reaching the accurate age of treating (fourth instar). The bioassay experiments were performed on the fourth instar larvae, because they represent the youth stage which causes massive crop damage and exhibits 72 resistance to the insecticides (El Sayed et al. 2022). During the bioassay experiments, the larvae were fed on castor bean leaves treated with the actinobacterial metabolic extracts for 48 h. Then replaced, with fresh castor bean leaves for the rest of their life until pupation. The commercial insecticide Radiant SC12%, was used as a positive control in the bioassay experiments. The detailed insect breeding protocol is shown in Additional file 1 (S1).

### Actinobacteria fermentation and metabolites extraction

Two µl of spore suspension (2–8 × 10^7^ cfu/mL) of each actinobacterial strain was cultured in 50 mL sterilized starch casein broth (Sigma-Aldrich, Chemie GmbH, Taufkirchen, Germany) and incubated at 28 ± 2 °C for 21 days with continuous shaking at 100 rpm. The mycelia were separated by centrifugation at 5000 rpm. The filtrates were extracted three successive times using equal volumes of ethyl acetate (El Nasr Pharmaceutical Chemicals Co., ADWIC, Egypt). The solvent layers were combined, concentrated, and evaporated to dryness using a rotary evaporator (HS-2005S-N, HAHN SHIN Scientific Co., Korea) at 40 °C. The dried extracts were redissolved in ethyl acetate to prepare a stock concentration of 100 mg/mL and stored at 4 °C for bioactivity screening tests.

### Screening effective metabolites for toxicity to fourth instar larvae of laboratory *Spodoptera littoralis* (L-larvae)

A series of four concentrations (0.6, 6, 60, 100 mg/mL) of crude metabolites, redissolved in ethyl acetate, from the seventy actinobacteria strains were prepared. Newly molted fourth instar laboratory, L-larvae, were starved for three-four hours prior to the treatment, to clear their alimentary canal and assure quick ingestion of treated leaves. Groups of larvae were transferred to 350 mL sterilized clean glass jars, and all jars were supplied with 7.0 cm filter paper to absorb any surplus moisture. Healthy, untreated leaves of castor, *Ricinus communis* L., were collected from the experimental field of the Plant Protection Research Institute. The leaves were washed, cut into equal discs using a cork borer, and impregnated with 50 µl of the corresponding metabolite concentration (i.e., equivalent to 0.03, 0.3, 3 and 5 µg/disc) using a leaf dipping technique. The toxicity was assessed in comparison to that of Radiant SC 12%, at LC50 0.5 mL/L (i.e., 0.05% concentration). All bioassay assessments were replicted (each replicate included four larvae), and were performed under constant laboratory conditions. The lethal effects (mortality %) were recorded, daily, and corrected according to Abbott's formula (Abbott 1925). The treated insects were followed up, until the pupation stage.

### Screening effective metabolites for toxicity to fourth instar larvae of field *Spodoptera littoralis* (F-larvae)

Metabolic extracts (from 7 strains) that showed the highest activity against L-larvae were selected for investigation with the field F-larvae, at 100 mg/mL concentration, as detailed above. The most potent strain *Streptomyces* sp. ES2 was, then, selected for detailed characterization and toxicity investigations. The toxicity was assessed in comparison to that of Radiant SC 12%, at LC_50_ 0.5 mL/L (i.e., 0.05% concentration). The experimental design included two control groups, fed on leaves treated with distilled water and ethyl acetate, respectively. All bioassay assessments were in triplicates (each replicate included four larvae), and were performed under constant laboratory conditions.

### Identification of the most active strain *Streptomyces* sp. ES2

*Streptomyces* sp. ES2 was cultivated on ISP 4 medium, Inorganic Salt Starch Agar (HiMedia, India), (Shirling and Gottlieb 1966). The strain was incubated at 28 ± 2 °C for 14 days, and Scanning electron microscopy (SEM) images of the sporulated hyphae was investigated using LEO GEMINI-1530 high-resolution electron microscope (Carl Zeiss, SMT GmbH, Oberchoken, Germany). For the Detection of Diaminopimelic acid (DAP) isomers, ES2 strain was grown in Tryptone Soya broth (TSB) at 100 rpm, 28 °C for seven days. The mycelia were harvested by centrifugation at 12,000 × g, washed twice with sterile distilled water and dried. Two mg of the dried mycelia were hydrolyzed in 1 mL 6N HCl, as described by Staneck and Roberts (1974). The hydrolysate was filtered and run in parallel to DAP (DL-diamonipimelic acid) and LL-DAP standard on a thin layer chromatography plate. The bands were developed using ninhydrin solution and drying at 80 °C. The cultivation and phenotypic characterization of *Streptomyces* sp. ES2 was performed in triplicates, to guarantee the quality of the results.

For partial 16S rRNA gene sequence analysis, DNA was extracted using the salting-out method (Kieser et al. 2000), with an additional purification step using phenol/chloroform. The 16S rRNA gene of the strain was amplified using the universal primer set 27F (5´-AGA GTT TGA TCC TGG CTC AG-3´) and 1492R (5´-GGT TAC CTT GTT ACG ACT T-3´), (Metabion International AG, Planegg, Germany). Amplification conditions were according to Trujillo et al. (2010). Briefly: an initial denaturation step was performed for 9 min at 94 °C, followed by 30 cycles of denaturation for 1 min at 95 °C, annealing for 1 min at 55 °C and extension for 2 min at 72 °C. A final extension step was performed for 10 min at 72 °C.

PCR product sequencing was performed at Macrogen Biotechnology, Ltd. (Korea) (https://dna.macrogen.com/eng/). The sequence obtained and those of its most closely related *Streptomycetes* spp., retrieved from GenBank, were aligned using BLASTN (Version: 2.9.0 +) (Zhang et al. 2000). The maximum identity score sequences were selected and aligned using the multiple alignment program ClustalW (Thompson et al. 1997). The phylogenetic tree was established by the maximum likelihood method, 1000 bootstrap, Tamura 3-parameter model; constructed using MEGA11 (Tamura et al. 2021).

### Histopathological examinations of L-larvae treated with ES2 metabolic extract

The efficacy testing of natural products for toxicity and mortality should be documented 48 and/or 72 h after exposure, according to the WHO standards for laboratory and field testing of insecticidal activity (Yadav 2013). This is due to the possibility that natural products contain substances with fundamentally novel mechanisms of action on insects. Therefore, samples of the treated L-larvae and controls were collected at 48 and 72 h post treatment. They were preserved in 3 mL 10% formaldehyde (v/v), (El Nasr Pharmaceutical Chemicals Co., ADWIC, Egypt), in sterilized screw-capped tubes, dehydrated and embedded in paraffin wax. Serial longitudinal and transverse sections, at five microns thickness, were made with a microtome and mounted on clean slides using Mayer’s albumin (Stanbio laboratory, India). The sections were stained with Ehrlich’s hematoxylin–eosin (HE), (TissuePro Technology, Gainesville, FL, USA), (Ruiz et al. 2004). The histological sections were examined under a light binocular stereomicroscope (NOVEL; NLCD-120, China) at 100-X and 400-X magnifications.

### Biochemical examinations of L-larvae treated with ES2 metabolic extract

Samples (groups of four) of the treated and control L-larvae were placed in clean screw-capped tubes and kept frozen overnight. The frozen samples were homogenized for three minutes in distilled water (50 mg/mL) using a chilled glass Teflon tissue homogenizer (ST–2 Mechanic-Preczyina, Poland) surrounded with a crushed ice jacket. Then, they were centrifuged at 8000 rpm for 15 min at 5 °C in a refrigerated microcentrifuge (Hettich, Kirchlengern, Germany). The supernatants, used as enzyme extracts, were stored at − 20 °C until use in biochemical assays. All biochemical measurements were performed in triplicates. A double beam UV spectrophotometer (Spectronic 1201, Milton Roy Co., Georgia, USA) was used to measure the absorbance of colored substances.

The total protein concentration was determined according to Bradford's method (Bradford 1976).

Acetylcholinesterase (AchE, EC 3.1.1.7) determination: acetylcholinesterase activity, a detoxification enzyme, was measured according to Simpson et al. (1964), using acetylcholine bromide (AchBr), (Sigma-Aldrich, Chemie GmbH, Taufkirchen, Germany), as a substrate. The samples were measured at 515 nm absorbance against a blank (ethanol in phosphate buffer, pH 8.0), (El Nasr Pharmaceutical Chemicals Co., ADWIC, Egypt). The activity was expressed as U/mg protein.

Protease (EC 3.4.21.112) determination: proteolytic activity was measured as described by Tatchell et al. (1972), with modifications, by measuring the increase in free amino acids split from a substrate protein (albumin) during one hour of incubation at 30 °C. Amino acids were colorimetrically assayed by ninhydrin reagent (Sigma-Aldrich, Chemie GmbH, Taufkirchen, Germany). The zero adjustment was performed at 570 nm against the reagent blank (100 µl distilled water). The amino acids were expressed as µg D, L-alanine/min/mg protein.

Lactate dehydrogenase (LDH, EC 1.1.1.27) determination: LDH activity was performed as described by Diamantino et al. (2001). The zero adjustment was performed against buffer without substrate. The activity was expressed as U/mg protein (1 U = 1 μmol substrate hydrolyzed per minute).

### Non targeted metabolomics analysis

Liquid chromatography, combined with quadrupole-time-of-flight high-definition mass spectrometry, LC-Q-TOF-MS, was used to investigate the chemical constituents of the metabolites from *Streptomyces* sp. ES2 strain. This technique is a powerful tool for the characterization of microbial compounds with similar structures, particularly in the analysis of natural products (Liu et al. 2010). The analysis was performed using Triple TOF^®^ 5600 + , Sciex system, Canada; pre-column (0.5 μm × 3.0 mm; Phenomenex Co., USA) and XBridge C18 column (3.5 μm, 2.1 × 50 mm; Waters Co., USA) with two LC columns, in-line filter discs, at 40 °C. Detailed preparation and processing of the sample is provided in the Additional file 1: (S2). Based on their fragments, MasterView was used to define peaks using Build-in databases (Data acquisition Analyst TF 1.7.1 software, Sciex). Reaxys ChemDraw software, version 18.0.0.20 (https://www.reaxys.com) was used to the compounds that can effectively target lethality to the larvae (Table 4).

### Molecular docking simulation

Molecular docking aimed to illustrate the virtual mechanism of binding of selected compounds which towards acetylcholinesterase (AchE, PDB = 4EY5), lactate dehydrogenase (LDH, PDB = 1LDG), and protease (SREBPs, PDB = 5GPD) target proteins. The data were freely accessible through the protein data bank. Both proteins and ligands were optimized, and the molecular docking study was carried out using AutoDock Vina as the computational software (Trott and Olson 2010). Each complex was analyzed for 3D interaction images taken by Chimera (UCSF) (Pettersen et al. 2004).

### Statistical analysis

All data were formulated as means ± standard error of the mean (SEM). The data wee subjected to normality testing using Kolmogorov–Smirnov at 0.05 level. Accordingly, LDH, protease and AchE were parametric and parametric data analysis applied. One-way ANOVA was applied to assess the difference between treatment groups, ANOVA was followed by Duncan’s Multiple Range tests (DMRTs) as a post-hoc test at 0.05 level.

## Results

### Toxicity of actinobacteria metabolites to *Spodoptera littoralis* larvae

Toxicity of the seventy actinobacteria metabolic extracts to *S. littoralis* fourth instar larvae was evaluated at 0.6, 6, 60, and 100 mg/mL concentrations. The screening has shown highest activities of seven actinobacteria on the laboratory strain (L-larvae); with five strains being toxic to the field strain (F-larvae). Using 100 mg/mL extracts, we observed significant and immediate death in the treated larvae 48 h after treatment. The latent effects varied between deaths of the larvae after five days of the treatment, to death of the resulted pupae (Table 2).

**Table 2:** Toxicity of the potent metabolic extracts to the fourth instar larvae of *Spodoptera littoralis,* laboratory and field strains, at concentration 100 mg/mL

Actinobacteria		Laboratory strain mortality		Field strain mortality	
Code	genus	Immediate ^a^	Latent ^b^	Immediate	Latent
11	*Nocardioides* sp.	+ + +	+	** + + **	+
19	*Nocardioides* sp.	+ + +	+ + +	** + + **	+
26	*Streptomyces* sp.	** + + + **	** + + + **	na	+ +
53	*Streptomyces* sp. ES2	** + + + **	+	** + + + **	+
62	*Streptomyces* sp.	** + + + **	** + + + **	na	na
67	*Pseudonocardia* sp.	** + + + **	** + **	na	na
68	*Streptomyces* sp.	** + + + **	na	na	+ +
Dist. Water Control		–	–	–	–
EtAc Solvent Control		na	na	na	na
Radiant SC 12% Control		** + + + **	na	** + + + **	na

^a^, death within 72 h; ^b^, late death of larval/pupal stages;

+  +  + , 50–30% mortality; +  + , 29–11% mortality; + , ≤ 10% mortality of pupal stage; na, no activity

Dist. Water Control: are *S. littoralis* larvae fed on leaves sprayed with distilled water; EtAc Solvent Control: are *S. littoralis* larvae fed on leaves sprayed ethyl acetate solvent. All bioassay assessments were duplicated (each replicate included four larvae)

**Fig. 1:**
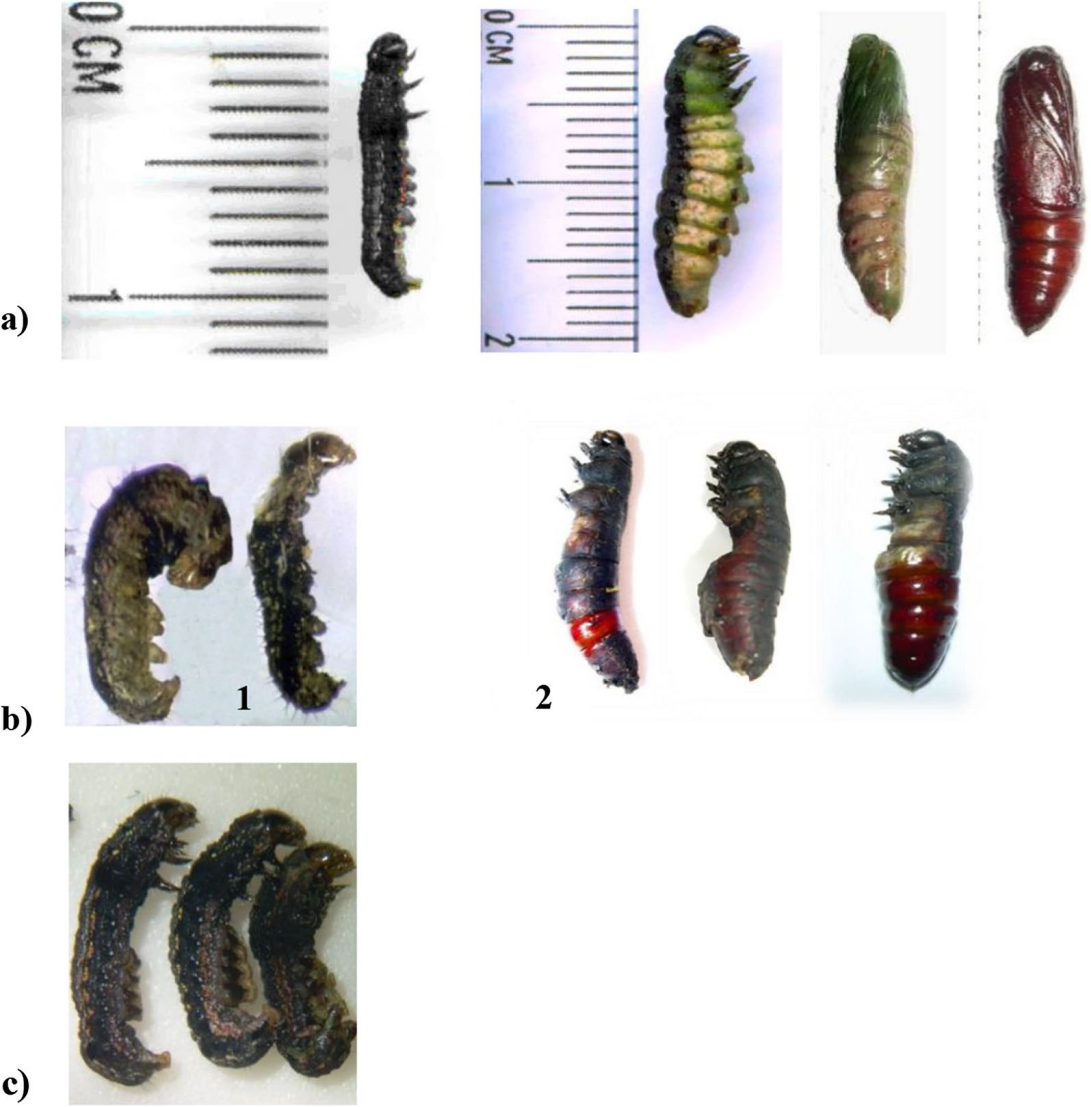
Lethality and developmental defects in fourth instar laboratory *Spodoptera littoralis* larvae after exposure to *Streptomyces* sp. ES2 metabolites. **a** Control *Spodoptera littoralis* (from left: larva, prepupa, young pupa and mature pupa). **b** Effects of the *Streptomyces* sp. ES2 metabolic extract; various direct toxicity on larvae (1) and latent effects on pupae (2). **c** Morphological effects of the commercial product Radiant SC 12% on larvae. All bioassay assessments were duplicated (each replicate included four larvae)

However, at metabolite concentrations below 100 mg/mL, biological effects such as deformed individuals (Fig. 1b1) and abnormalities in pupae (Fig. 1b2, c) were observed and described as latent toxic effects, compared to the controls (Fig. 1a). The detailed toxic effects of the seventy metabolites on the L-larvae, at 100 mg/mL concentration, are shown in Additional file 1: Table S3.

### Identification of the strain *Streptomyces* sp. ES2 EMCC2291

**Fig. 2:**
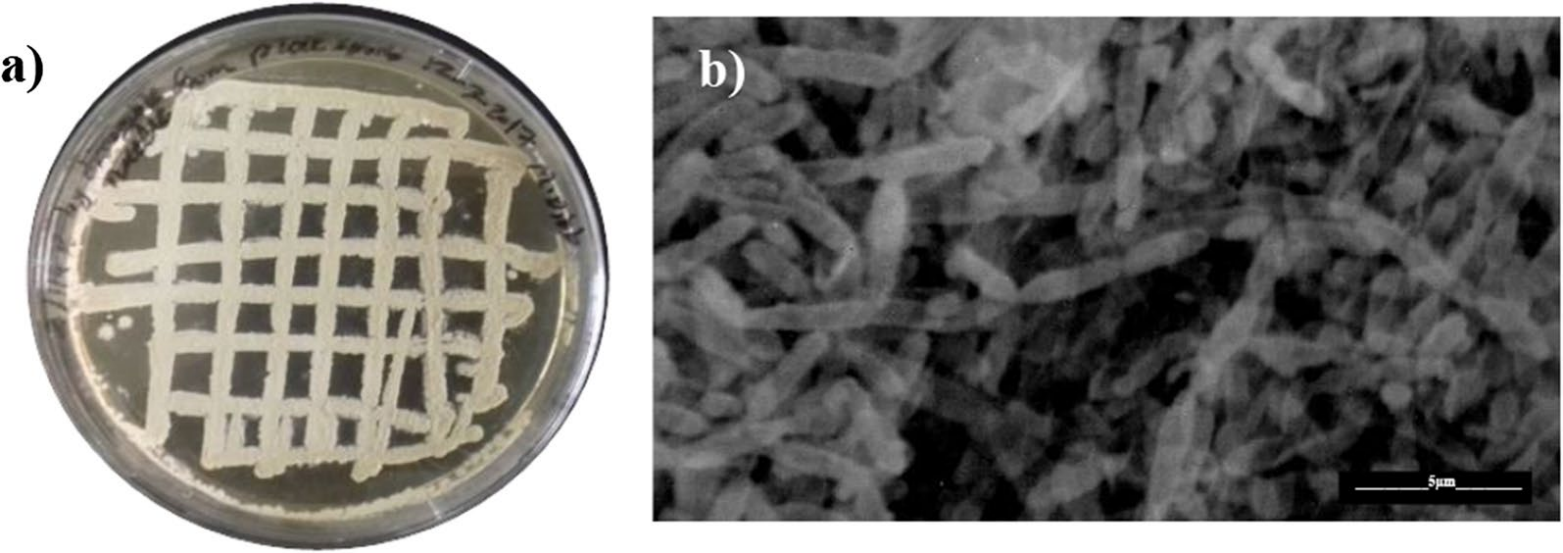
Macroscopic view **a** and scanning electron microphotograph **b** of *Streptomyces* sp. ES2 after cultivation on ISP 4 medium (Inorganic Salt Starch Agar), at 28 °C for 15 days. The photograph of the spores was taken by SEM. Bar 5 µm

Among the seven actinobacteria isolates showing inhibitory effect on the cotton leafworm, we have selected *Streptomyces* sp. ES2, based on its potent effect. Figure 2 shows the micromorphology of strain ES2 grown on ISP 4 medium. The partial 16S rRNA gene sequencing has shown its closest similarity to *Streptomyces* spp*.*, as shown in the phylogenetic tree (Additional file 1: Fig. S1). The 16S rRNA gene sequence of strain ES2 is deposited in the GenBank database under the number (MH200991). The culture is deposited at Cairo MIRCEN Culture Collection, with the ID: ES2 EMCC2291, and the details related to ES metabolite production is submitted for patency at the Egyptian Patent Office, patent No. 729/2019.

### Detailed toxicity of ES2 metabolite to the larvae

**Fig. 3:**
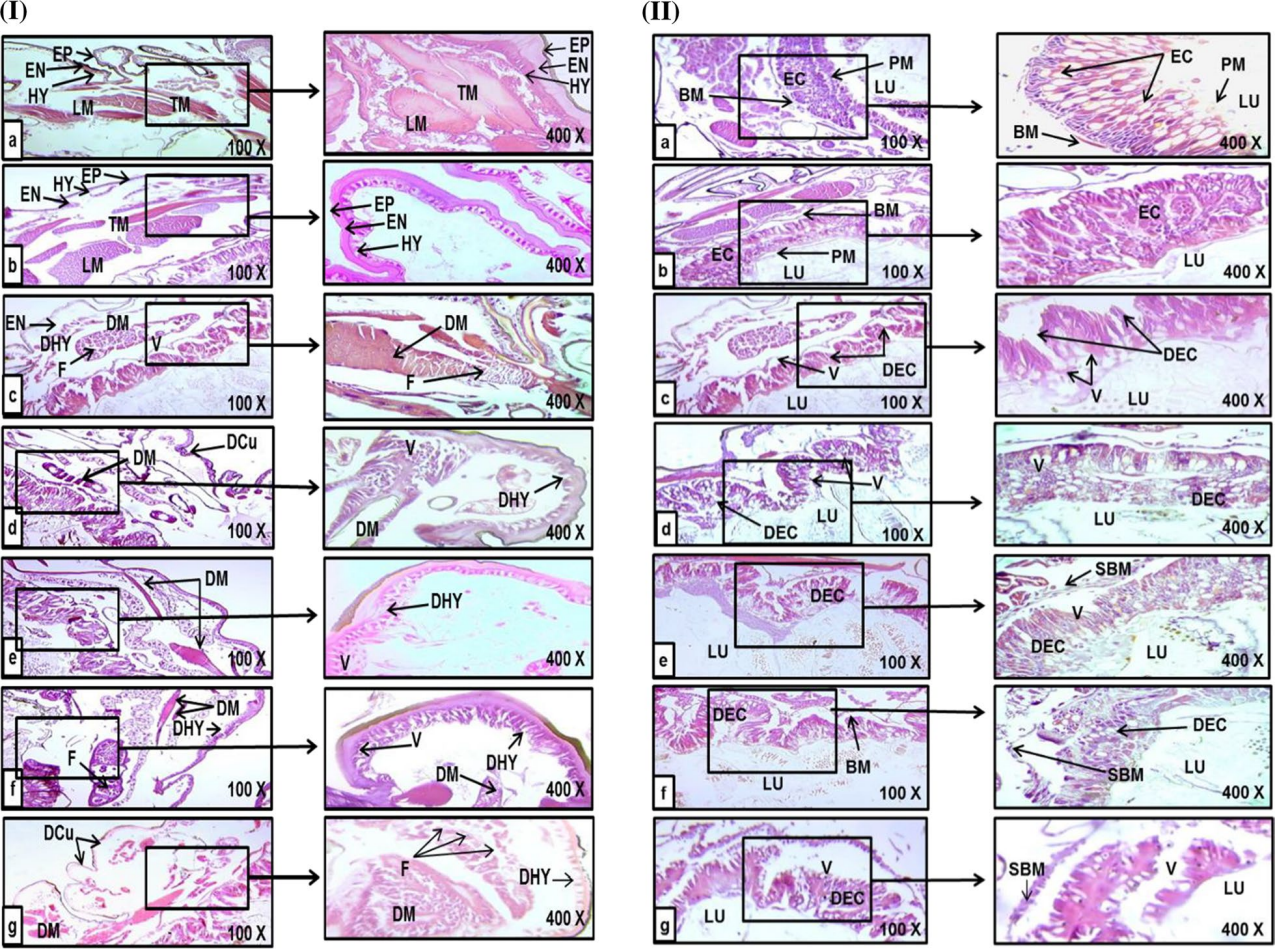
Light micrographs of (1) longitudinal and (2) transverse sections of histopathological deformities on the *Spodoptera littoralis* larval cuticle & midgut tissue, three days post treatment with *Streptomyces* sp. ES2 crude metabolite, showing muscle deformities (100 & 400 × H & E). **a** Control larvae treated with water; **b** Negative control larvae treated with ethyl acetate solvent; **c**–**f** Larvae treated with the *Streptomyces* sp. ES2 crude metabolite; and **g** Positive control larvae treated with Radiant SC 12%. [DCu, degenerated cuticle; DHY, deformed hypodermis; DM, degenerated muscles; EN, endocuticle; EP, epicuticle; F, fissures in muscles; HY, hypodermis; LM, longitudinal muscles; TM, transverse muscles; V, vacuoles, BM, basement membrane; DEC, degenerated epithelial cells; EC, epithelial cells; LU, lumen; PM, peritrophic membrane; SBM, separated basement membrane; V, vacuoles]. All histopathological examinations were duplicated per each treatment (each replicate included four larvae)

*Streptomyces* sp. ES2 has caused 50% death in the L-larvae after 48 h post-treatment. Also, some toxic effects have appeared in the treated larvae after 72 h; those were considered latent effects. It is noteworthy that, some larvae failed to pupate from the larval stage to the pupal stage, leading to the production of larval-pupal or pupal-larval intermediates, as shown in (Fig. 1). The cuticle layers were distorted in the larvae treated with both ES2 (Fig. 3.I,d) and Radiant (Fig. 3.I, g), compared to the untreated control (Fig. 3.I, a, b). Larvae that were treated with the ES2 have shown obviously higher degeneration and fissures in the muscles (Fig. 3.I, c, d, e, f), than those subjected to the Radiant treatment (Fig. 3.I, g). ES2 has, also, caused deformations (Fig. 3.I, c, d, e, f), vacuolization of the hypodermal layer (Fig. 3.I, d, e, f), and separation of the hypodermis from the cuticle layer (Fig. 3.I, c, e). However, decomposition in the hypodermal cells of occurred only in the larvae treated with Radiant SC 12% (Fig. 3.I, g). ES2 has caused obvious gastrointestinal damages in the larval midgut tissue of *Spodoptera littoralis*, as compared to the control (Fig. 3.II, a, b). These damages included: vacuolization (Fig. 3.II, c, d, e), degeneration and necrosis of the epithelial cells and destruction of the cells and their boundaries (Fig. 3.II, c, d, e, f). The microscopically observed changes were more severe than those observed in the Radiant-treated larvae (Fig. 3.II, g). Separations of the basement and peritrophic membranes were, similarly, observed for both ES2 (Fig. 3.II, e, f) and Radiant (Fig. 3.II, g) treatments.

The biochemical measurements, in our study, served to provide a preliminary understanding of the basis of ES2 toxicity. Where the application of ES2 and Radiant at 100 and 5 mg/mL, respectively, has caused highly significant reductions in lactate dehydrogenase (LDH) and protease activities (p ≤ 0.01). While acetylcholinesterase (AchE) activity was not significantly changed, compared to the control. LDH activity in the untreated (control) larvae was 28.40 ± 0.888 U/mg protein, and it has been reduced by 47% and 16%, using ES2 and Radiant treatments, respectively. The reductions in protease activity were almost similar after both treatments (37.00 ± 2.081 and 30.33 ± 0.881 µg D, l-alanine/min/mg protein for ES2 and Radiant, respectively), as shown in Table 3.

**Table 3:** Lactate dehydrogenase (LDH), protease and acetylcholinesterase (AchE) activities of the laboratory *S. littoralis* treated with *Streptomyces* sp. ES2 crude metabolite

Treatment	LDH *(U* × *10*^*3*^*/mg protein)*	Protease *(ug D,L-alanine/min/mg protein)*	AchE *(ug AchBr/min/mg protein)*
Normal distilled water	28.4 ± 0.89 a	101.66 ± 3.84 a	2.40 ± 0.09 a
Negative Control Ethyl acetate	26.42 ± 0.68 a	92.33 ± 1.45 b	2.15 ± 0.09 a
Radiant SC 12% (0.05%)	23.8 ± 0.62 b	30.33 ± 0.88 c	2.31 ± 0.06 a
ES2 Metabolite (100 mg/mL)	15.06 ± 0.54 c	37.00 ± 2.08 c	2.16 ± 0.05 a
ANOVA- 1 way			
F-ratio	72.25	247.08	2.69
*p*-value	< 0.001***	0.001***	> 0.05 NS

Data expressed as Mean ± S. E Mean. Different letters (a, b & c) in the same column denote a significant different between groups, according to DMRTs, at p ≤ 0.05. *** = p ≤ 0.01 NS = non-Significant

Normal, Control treated with distilled water for experiment adjustment; Negative Control, control treated with ethyl acetate solvent for experiment adjustment; ES2 Metabolite, the most potent actinobacterial crude metabolite produced by *Streptomyces* sp. ES2 strain; F-ratio, Frequency ratio

All biochemical measurements were performed in triplicates

### Structure analysis and molecular docking simulation of ES insecticidal activity

**Fig. 4:**
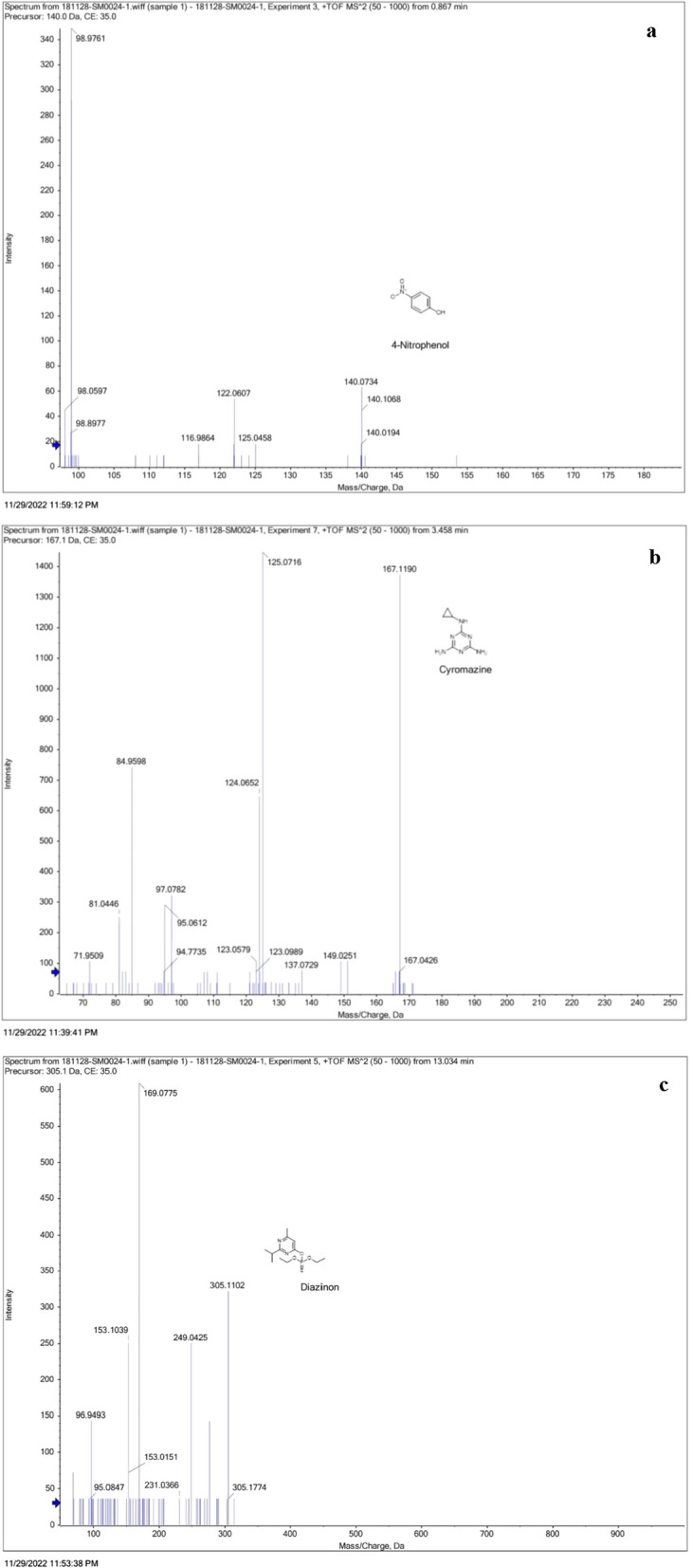
LC-Q-TOF-MS spectra with fragmentation peaks of the compounds **a** 4-nitrophenol, **b** cyromazine and **c** diazinon, at m/z 140.1, 167.1 and 305.1, respectively. Mass chart was shown as m/z values relative to signal abundance (%). Beaks with the highest abundance are the molecular ion peak

MS analysis showed three insecticidal compounds as key constituents of the metabolic extract from *Streptomyces* sp. ES2, 4-nitrophenol, cyromazine and diazinon, as shown in Table 4. The chromatogram in Additional file 1: Fig. S2 shows the positive mode–Base Peaks Compounds (BPC). The identified peaks of the three compounds: 4-nitrophenol, cyromazine and diazinon are highlighted (Fig. 4 and Table 4), showing the [M + H] “molecular weight” for each fragment, and the intensity for each. The pharmacophoric regions aromatic, polar, and nonpolar moieties) of the three compounds are highlighted in the Additional file 1: Fig. S3. They were docked and visualized inside the acetylcholinesterase (AchE), lactate dehydrogenase (LDH), and protease proteins to highlight their virtual mechanism of binding in terms of binding energy and interactions.

**Table 4:** Compounds with insecticidal activities identified in ethyl acetate extract of *Streptomyces* sp. ES2 EMCC2291 by LC-QTOF-MS–MS technique

Identified compound	RT (min.)	Intensity	Mass (Da)	Adduct	m/z value (mass)	Molecular formula	InChI Key	Chemical structure	ChemSpider ID	KEGG ID	PubChem CID	METLIN ID
4-Nitrophenol	0.9714333	12436.39	139.1088	[M + H]^+^	140.1089	C_6_H_5_NO_3_	BTJIUGUIPKRLHP-UHFFFAOYSA-N		955	C00870	980	4100
Cyromazine	3.467633	56928.55	166.09669	[M + H]^+^	167.1178	C_6_H_10_N_6_	LVQDKIWDGQRHTE-UHFFFAOYSA-N		43550	C14147	47866	NA
Diazinon	13.02	1941	304.10105	[M + H]^+^	305.10833	C_12_H_21_N_2_O_3_PS	FHIVAFMUCKRCQO-UHFFFAOYSA-N		2909	C14324	3017	NA

RT (min,); Retention time in minutes, Mass (Da); molecular weight in Dalton. (m/z) are values detected by mass spectroscopy. Compounds were retrieved from PubChem databases and drawn by ChemDraw software

Chemspider ID, Chemical structure Database Identifier (http://www.chemspider.com/); InChI Key, International Chemical Identifier (https://pubchem.ncbi.nlm.nih.gov/source/ChEBI); KEGG ID, Kyoto Encyclopedia of Genes and Genomes database (https;//www.genome.jp/kegg/)

**Fig. 5:**
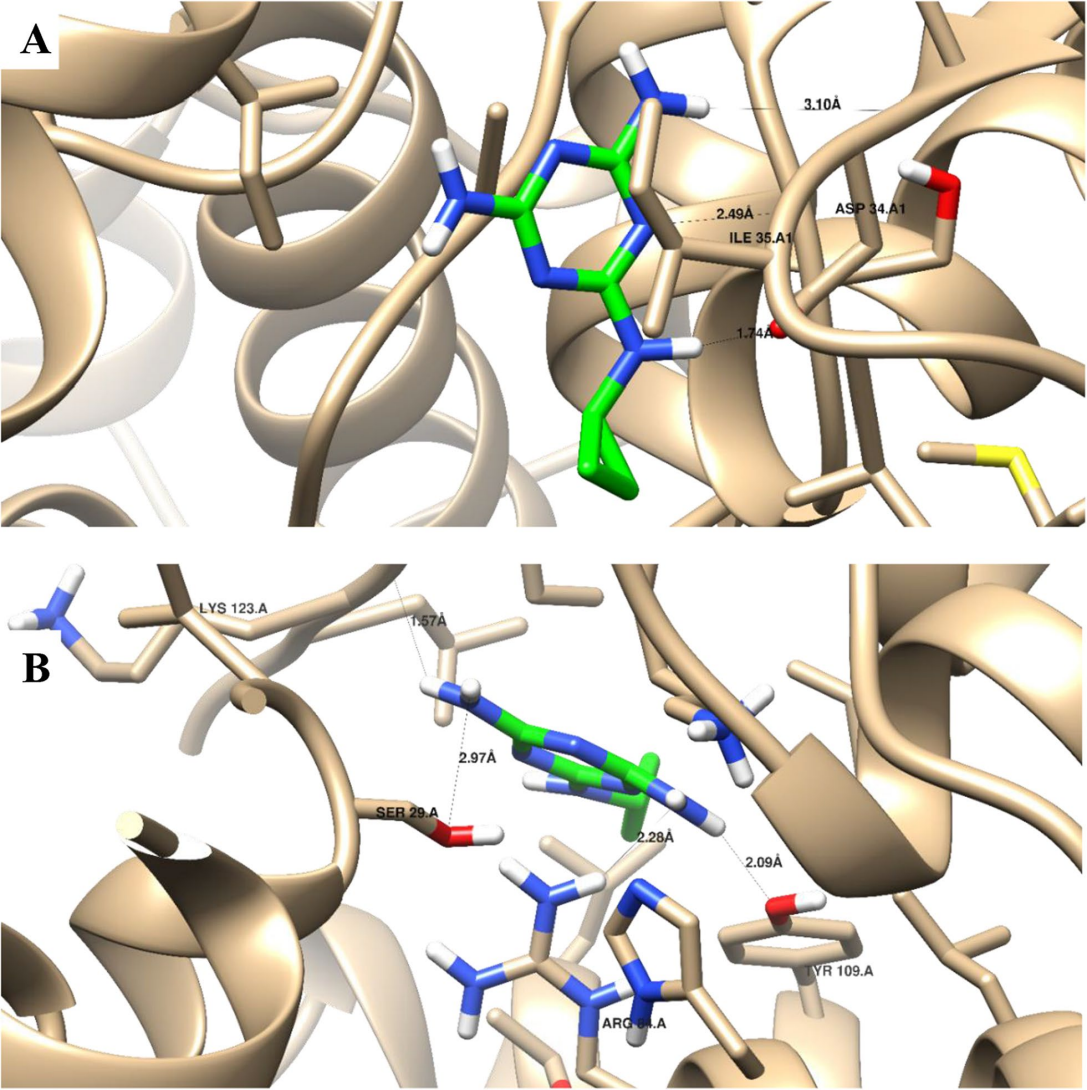
Binding disposition and ligand-receptor interactions of Cyromazine as Green-colored with promising insecticide activity towards **A** lactate dehydrogenase (LDH), and **B** protease (SREBPs) active sites as Buff-colored. Heretoatoms of Oxygen, Nitrogen, Hydrogen with standard colors. Labeled amino acids are the highlighted key amino acids for interaction. H-Bond distances were calculated in Angstrom (Å). These tested proteins were chosen following the experimental biological examinations

Molecular docking for 4-nitrophenol and diazinon is summarized in Table 5. It shows proper docking inside the active sites of LDH and protease, with good binding energies. Both compounds formed strong interactions with the key amino acids of LDH (Asp 53 and Gly 99), and protease (Arg 740). While, a weak binding affinity was found towards AchE. The compounds form interactions with amino acids other than the key interactive ones. A weak interactions is observed with the key amino acid (Trp 86) inside AchE protein. As seen in Fig. 5, cyromazine formed good binding interactions through its active pharmacophoric groups with the key amino acids of the LDH and protease proteins. The molecular docking studies, agreed with the experimental results enhancing the idea of their insecticidal activity through lactate dehydrogenase, and protease inhibition.

**Table 5:** Molecular docking results for two insecticidal compounds (4-nitrophenol and diazinon) as key constituents of the metabolic extract from *Streptomyces* sp. ES2 EMCC2291 insides AchE, LDH, and Protease proteins

	4-nitro phenol		Diazinon	
	Binding energy (Kcal/mol)	Ligand-recptor Interactions	Binding energy (Kcal/mol)	Ligand-recptor Interactions
Acetylcholinesterase (AchE)	− 2.3	1 H-bond with Tyr 133 Vanderwaal forces with **Trp 86**	− 3.4	3 H-Bonds with Tyr 124, Tyr 337, and Ser 125 Vanderwaal forces with **Trp 86**
Lactate dehydrogenase (LDH)	− 12.3	1 H-bond with **Asp 53**	− 15.6	1 H-bond with **Gly 99**
Protease	− 16.4	1 H-bond with **Arg 740**	− 16.7	4 H-bonds with Ser 685, Lys 784, **Arg 740**

Bold amino acids are the key interactive ones with which that native ligands interact. Binding energy of ligand-protein complex is expressed as Kcal/mol

Gly 99, dipeptide Glycyl-glycine metabolism; Arg 740, Arginine amino acid metabolism; Asp 53, aspartic acid metabolism; Trp 86, Tryptophan amino acid metabolism

## Discussion

The lepidopterous pest *Spodoptera littoralis* has developed resistance due to the intensive pesticide use (Rehan and Freed 2014), imposing the need for novel pesticide compounds. This study aimed to evaluate the insecticidal effects of the secondary metabolites of actinobacteria strains from the microbiome of six medicinal plant species in the World Heritage Site of Saint Catherine, Egypt.

Notably, all the actinobacteria strains originated from *Artemisia herba-alba* and *Artemisia judaica* plants, have shown high toxicity to the fourth instar larvae of *Spodoptera littoralis*. Giving the evidence that endophytes can produce the same bioactive chemicals as their host plants (Toghueo and Boyom 2019), this study emphasizes the insecticidal activity of the microbiome from *Artemisia* spp.

The partial 16S rRNA gene sequence demonstrated close relation of the most potent actinobacterium ES2 to *Streptomyces* spp. It should be highlighted that the strain ES2 closely resembles *Kitasatospora* spp. according to our previous chemotaxonomic analysis (El-Shatoury et al. 2013). Given the challenges in clearly differentiating between the genera *Streptomyces* and *Kitasatospora*, the present non-matching result is predicted. Though the two genera have genetic similarities, the cell walls of *Kitasatospora* differs in that it includes both LL- and meso-diaminopimelic acid, glycine, and galactose (Takahashi 2017). We are currently performing multilocus phylogenetic analysis, data not published, to confirm the taxonomic position of the strain *Streptomyces* sp. ES2.

The results indicated significantly strong effect of ES2 metabolite in LDH inhibition. LDH is an important glycolytic enzyme involved in carbohydrate metabolism and is a marker for reduced metabolism in the insect (Diamantino et al. 2001). Our result compares well with other studies on the LDH inhibition by natural microbial products. For example, Nathan et al. (2006) demonstrated 20% suppression of the LDH activity in the fourth larval instar *Cnaphalocrocis medinalis* (the rice leaf folder), after exposure to 3 μg/mL of *Bacillus thuringiensis* bioinsecticide. Another field experiment showed 55.7% LDH inhibition, when the fifth instar larvae of *Spodoptera littoralis* were exposed to Radiant, at concentration 40 L/feddan (Fahmy and Dahi 2009).

Our results, also, indicated highly significant inhibitory effect of both ES2 and Radiant on protease (64–70% inhibition). Proteases are involved in the glycolysis process and the neural signals transmission in insects (Cheng et al. 1999). In light of this, we propose that ES2 metabolite may affect insect metabolism and result in mortality by interfering with glycolysis as a result of the inhibition of both LDH and protease enzymes. In contrast to the field strain, the laboratory strain was more sensitive to ES2 metabolites. Field strains generate high levels of resistance to pesticides due to a variety of mechanisms, including metabolic detoxification of insecticides or hereditary resistance to the insecticide (Fahmy and Dahi 2009; Krestonoshina et al. 2022).

Our findings show that, ES2 does not appear to target the neurological system since it has induced insignificant alterations in acetylcholinesterase activity, in contrast to Radiant, which causes death owing to disruption of the nervous system (Krämer and Schirmer 2007). The acetylcholinesterase is crucial for the transmission of nerve messages in the insect's body (Casida and Durkin 2013). Therefore, ES2 could operate in a different manner than Radiant.

It is possible to relate the insect's toxicity, morphological flaws, and histological malformations to the unique blend of constituents in ES2 metabolite; with cyromazine serving as a significant element. Cyromazine has long been used to control pests in agricultural crops and public health management systems (Subramanian and Shankarganesh 2016). It is has a molt inhibitor effect, since it interferes with cuticle production in the insect (Pener and Dhadialla 2012). This effect on cuticle, perhaps, was responsible for the symptoms of mortality in *Spodoptera littoralis* fourth instar larvae in our investigations. This notion is supported by morphological and histological examinations of midgut tissue. Where, the muscular and gastrointestinal damages caused by ES2 metabolite were more severe than those produced by Radiant.

Feeding on ES2 may have, also, hampered growth since the body wall was unable to expand to accommodate the increasing body mass. The observed lesions in the body wall cuticle might be the result of severe internal pressure. Furthermore, locomotion may have been hampered because high pressure in the haemolymph hinders the caterpillar's hydrostatic skeleton from functioning normally. These symptoms are thought to be responsible for the observed larval mortality and the creation of intermediate individuals in our experiment.

Recent studies which characterize secondary metabolites from actinobacteria, shows insect growth disrupting activities against various species of *Spodoptera*. These include polyketides that inhibit insect growth (Arasu et al. 2013), juvenile hormone antagonists (Kim et al. 2020) and chitin synthesis inhibitors (Usuki et al. 2008). However, this is the first report on the biological production of cyromazine by actinobacteria.

Despite similarities in their effects on Protease and AchE activities, ES2 metabolite and Radiant are thought to have distinct modes of action for a couple of reasons. First, unlike the spinosyn class (Crouse et al. 2001), ES2 is moderately polar and water-soluble, which is a major and promising distinction for future applications. Second, unlike Radiant, ES2 did not exhibit neuron overexcitation signs (Orr et al. 2009). By using molecular docking, we have validated the experimental results of the metabolite ES2's insecticidal effect through acetylcholinesterase, lactate dehydrogenase, and protease inhibition. The fractionation of the ES2 constituents is in progress to determine the active compounds and whether synergistic effects exist between the constituents.

In conclusion, our research demonstrates that endophytes inhabiting wild medicinal plants are a prospective source of natural products to control the cotton leafworm. Five endophytic actinobacteria strains are of great interest because of their toxicity to the Field strain of S. littoralis. Metabolites from *Streptomyces* sp. ES2 has shown direct and latent effects on the fourth instar larvae of the cotton leafworm *S*podoptera *littoralis*. We have used non-targeted metabolomic analysis to prove the existence of specific chemical compounds in ES2 product, and performed molecular docking to illustrate their virtual inhibitory mechanism. The toxicity of ES2 to *Spodoptera littoralis*, and the morphological defects and histopathological deformities may be attributed to its unique mixture of several compounds with special regard to cyromazine (a molt inhibitor), 4-nitrophenol, and diazinon as major constituents.

This is the first illustration of insecticidal activity of the *Artemisia* spp. microbiome, and the first report on a biological cyromazine synthesized by actinobacteria. Our take-home message from this work is that: With the shortage of natural pesticides on the market, endophytic actinobacteria appear as attractive eco-friendly pest management solutions. In our next publication, we anticipate reporting the complete chemical properties of the “active ingredients” produced by *Streptomyces* sp. ES2 and their expected targets, based on bioinformatic and molecular docking studies.

## Supplementary Information

**Additional file 1: Figure S1.** Phylogenetic tree of the strain* Streptomyces *sp. ES2 and the most related type strains, based on partial 16S rRNA gene sequences. **Figure S2.** Positive mode – Base Peaks Compounds (BPC) of ethyl acetate extract for *Streptomyces *sp. ES2 crude metabolite using LC-QTOF-MSMS. Chromatogram was shown as intensity relative retention time. **Figure S3.** Highlighted pharmacophoric regions (aromatic, polar, and nonpolar moieties) for the compounds with reported insecticide activities. **Table S3.** Lethal effects of actinobacteria crude extracts on the fourth instar larvae of laboratory *Spodoptera littoralis* (L-larvae).

## Data Availability

Partial 16S rRNA gene sequence of *Streptomyces* sp. ES2 is deposited in the GenBank database (No. MH200991).

## References

[CR1] Abbott WS (1925). A method of computing the effectiveness of an insecticide. J Econ Entomol.

[CR2] Alblooshi AA, Purayil GP, Saeed EE, Ramadan GA, Tariq S, Altaee AS, El-Tarabily KA, AbuQamar SF (2022). Biocontrol potential of endophytic actinobacteria against *Fusarium solani*, the causal agent of sudden decline syndrome on date palm in the UAE. J Fungi.

[CR3] Arasu MV, Al-Dhabi NA, Saritha V, Duraipandiyan V, Muthukumar C, Kim S-J (2013). Antifeedant, larvicidal and growth inhibitory bioactivities of novel polyketide metabolite isolated from *Streptomyces* sp. AP-123 against *Helicoverpa armigera* and *Spodoptera litura*. BMC Microbiol.

[CR4] Bachrouch O, Ferjani N, Haouel S, Jemâa JM, Ben, (2015). Major compounds and insecticidal activities of two Tunisian *Artemisia* essential oils toward two major coleopteran pests. Ind Crops Prod.

[CR5] Barka EA, Vatsa P, Sanchez L, Gaveau-Vaillant N, Jacquard C, Meier-Kolthoff JP, Klenk HP, Clément C, Ouhdouch Y, van Wezel GP (2016). Taxonomy, physiology, and natural products of *Actinobacteria*. Microbiol Mol Biol Rev.

[CR6] Bird LJ, Drynan LJ (2023). Comparison of insecticide toxicity in adult and larval stages of Spodoptera frugiperda (J.E Smith) and Helicoverpa armigera (Hünber) (Lepidoptera: Noctuidae). Crop Protection.

[CR7] Bradford MM (1976). A rapid and sensitive method for the quantitation of microgram quantities of protein utilizing the principle of protein-dye binding. Anal Biochem.

[CR8] Casida JE, Durkin KA (2013). Anticholinesterase insecticide retrospective. Chem Biol Interact.

[CR9] Chang Y-W, Wang Y-C, Yan Y-Q, Wu C-D, Xie H-F, Gong W-R, Du Y-Z (2022). Insect hormones affect the toxicity of the insecticidal growth regulator cyromazine in *Liriomyza trifolii* (Diptera: Agromyzidae). Pestic Biochem Physiol.

[CR10] Cheng D, Espenshade PJ, Slaughter CA, Jaen JC, Brown MS, Goldstein JL (1999). Secreted site-1 protease cleaves peptides corresponding to luminal loop of sterol regulatory element-binding proteins. J Biol Chem.

[CR11] Crouse GD, Sparks TC, Schoonover J, Gifford J, Dripps J, Bruce T, Larson LL, Garlich J, Hatton C, Hill RL, Worden TV, Martynow JG (2001). Recent advances in the chemistry of spinosyns. Pest Manag Sci.

[CR12] Deb MK, Dolly, (2020). Bioactivity and efficacy of essential oils extracted from Artemisia annua against Tribolium casteneum (Herbst. 1797) (Coleoptera: Tenebrionidae): an eco-friendly approach. Ecotoxicol Environ Saf.

[CR13] Diamantino TC, Almeida E, Soares AMVM, Guilhermino L (2001). Lactate dehydrogenase activity as an effect criterion in toxicity tests with *Daphnia magna straus*. Chemosphere.

[CR14] El Sayed YA, Sayed S, Magdy A, Elmenofy W (2022). Detection, characterization and virulence analysis of nucleopolyhedrovirus isolated from the cotton leafworm, Spodoptera littoralis (Boisd.) (Lepidoptera: Noctuidae). Egypt J Biol Pest Control.

[CR15] El-Shatoury SA, El-Kraly OA, Trujillo ME, El-Kazzaz WM, El-Din E-SG, Dewedar A (2013). Generic and functional diversity in endophytic actinomycetes from wild Compositae plant species at South Sinai-Egypt. Res Microbiol.

[CR16] El-Tarabily KA, Sivasithamparam K (2006). Non-streptomycete actinomycetes as biocontrol agents of soil-borne fungal plant pathogens and as plant growth promoters. Soil Biol Biochem.

[CR17] El-Tarabily KA, Nassar AH, Hardy GESJ, Sivasithamparam K (2009). Plant growth promotion and biological control of *Pythium aphanidermatum*, a pathogen of cucumber, by endophytic actinomycetes. J Appl Microbiol.

[CR18] El-Tarabily KA, AlKhajeh AS, Ayyash MM, Alnuaimi LH, Sham A, ElBaghdady KZ, Tariq S, AbuQamar SF (2019). Growth promotion of *Salicornia bigelovii* by *Micromonospora chalcea* UAE1, an Endophytic 1-aminocyclopropane-1-carboxylic acid Deaminase-producing Actinobacterial isolate. Front Microbiol.

[CR19] El-Tarabily KA, Ramadan GA, Elbadawi AA, Hassan AH, Tariq S, Ghazal EW, Abo Gamar MI, AbuQamar SF (2021). The marine endophytic polyamine-producing *Streptomyces mutabilis* UAE1 isolated from extreme niches in the Arabian Gulf promotes the performance of mangrove (*Avicennia marina*) seedlings under greenhouse conditions. Front Mar Sci.

[CR20] Fahmy M, Dahi H (2009). Changes in detoxifying enzymes and carbohydrate metabolism associated with spinetoram in two field-collected strains of Spodoptera littoralis (Biosd.). Egypt Acad J Biol F Toxicology Pest Control.

[CR21] Hazaa M, Alm-Eldin M, Ibrahim A-E, Elbarky N, Salama M, Sayed R, Sayed W (2020). Biosynthesis of Silver Nanoparticles using *Borago officinslis* leaf extract, characterization and larvicidal activity against cotton leaf worm.

[CR22] Hilliou F, Chertemps T, Maïbèche M, Le Goff G (2021). Resistance in the Genus *Spodoptera*: key insect detoxification genes. Insects.

[CR23] Kieser T, Bibb MJ, Buttner MJ, Chater KF, Hopwood DA (2000). Practical *Streptomyces* genetics.

[CR24] Kim HJ, White-Phillip JA, Ogasawara Y, Shin N, Isiorho EA, Liu H-w (2010). Biosynthesis of spinosyn in *Saccharopolyspora spinosa*: synthesis of Permethylated Rhamnose and characterization of the functions of SpnH, SpnI, and SpnK. J Am Chem Soc.

[CR25] Kim JH, Choi JY, Park DH, Park D-J, Park MG, Kim SY, Ju YJ, Kim JY, Wang M, Kim C-J, Je YH (2020). Isolation and characterization of the insect growth regulatory substances from actinomycetes. Comp Biochem Physiol Part C Toxicol Appl Pharmacol.

[CR26] Krämer W, Schirmer U (2007). Modern crop protection compounds, 3 set.

[CR58] Krestonoshina K (2022). Insect resistance to insecticides and approaches to its identification. Entomol Appl Sci Lett.

[CR27] Kvakkestad V, Sundbye A, Gwynn R, Klingen I (2020). Authorization of microbial plant protection products in the Scandinavian countries: a comparative analysis. Environ Sci Policy.

[CR28] Liu CW, Lu YY, Yang ZZ, Xing YY, Xi T (2010). Rapid screening and characterization of metabolites from a marine-derived actinomycete by high-performance liquid chromatography coupled with electrospray ionization quadrupole time-of-flight mass spectrometry. Rapid Commun Mass Spectrom.

[CR29] Mansour NA, Eldefrawi ME, Toppozada AR, Zeid M (1966). Toxicological studies on the Egyptian cotton leaf worm, *Prodenia litura*. VI. Potentiation and antagonism of Organophosphorus and Carbamate insecticides. J Econ Entomol.

[CR30] Nathan SS, Kalaivani K, Murugan K (2006). Effect of biopesticides on the lactate dehydrogenase (LDH) of the rice leaffolder, *Cnaphalocrocis medinalis* (Guenée) (Insecta: *Lepidoptera*: Pyralidae). Ecotoxicol Environ Saf.

[CR31] Orr N, Shaffner A, Richey K, Crouse G (2009). Novel mode of action of spinosad: receptor binding studies demonstrating lack of interaction with known insecticidal target sites. Pestic Biochem Physiol.

[CR32] Pasiecznik NM, Smith IM, Watson GW, Brunt AA, Ritchie B, Charles LMF (2005). CABI/EPPO distribution maps of plant pests and plant diseases and their important role in plant quarantine. Bulletin OEPP.

[CR33] Pener MP, Dhadialla TS, Dhadialla TS (2012). Chapter one—an overview of insect growth disruptors; applied aspects. Advances in insect physiology.

[CR34] Pettersen EF, Goddard TD, Huang CC, Couch GS, Greenblatt DM, Meng EC, Ferrin TE (2004). UCSF Chimera—a visualization system for exploratory research and analysis. J Comput Chem.

[CR35] Rang J, He H, Yuan S, Tang J, Liu Z, Xia Z, Khan TA, Hu S, Yu Z, Hu Y, Sun Y, Huang W, Ding X, Xia L (2020). Deciphering the metabolic pathway difference between *Saccharopolyspora pogona* and *Saccharopolyspora spinosa* by comparative proteomics and metabonomics. Front Microbiol.

[CR36] Rehan A, Freed S (2014). Selection, mechanism, cross resistance and stability of spinosad resistance in *Spodoptera litura* (Fabricius) (Lepidoptera: Noctuidae). Crop Prot.

[CR37] Ruiz LM, Segura C, Trujillo J, Orduz S (2004). In vivo binding of the Cry11Bb toxin of Bacillus thuringiensis subsp. medellin to the midgut of mosquito larvae (Diptera: Culicidae). Mem Inst Oswaldo Cruz.

[CR38] Shi Y, Zhang X, Lou K (2013). Isolation, characterization, and insecticidal activity of an endophyte of drunken horse grass. *Achnatherum inebrians*. J Insect Sci.

[CR39] Shirling EB, Gottlieb D (1966). Methods for characterization of *Streptomyces* species1. Int J Syst Evol Microbiol.

[CR40] Siddique S, Syed Q, Adnan A, Qureshi FA (2014). Isolation, characterization and selection of avermectin-producing *Streptomyces avermitilis* strains from soil samples. Jundishapur J Microbiol.

[CR41] Simpson DR, Bull DL, Lindquist DA (1964). A Semimicrotechnique for the estimation of cholinesterase activity in boll weevils1. Ann Entomol Soc Am.

[CR42] Sparks TC, Crouse GD, Benko Z, Demeter D, Giampietro NC, Lambert W, Brown AV (2021). The spinosyns, spinosad, spinetoram, and synthetic spinosyn mimics—discovery, exploration, and evolution of a natural product chemistry and the impact of computational tools. Pest Manag Sci.

[CR43] Staneck JL, Roberts GD (1974). Simplified approach to identification of aerobic actinomycetes by thin-layer chromatography. Appl Microbiol.

[CR44] Subramanian S, Shankarganesh K, Pest Ecofriendly (2016). Chapter 20 - Insect Hormones (as Pesticides). Omkar Management for Food Security.

[CR45] Takahashi Y (2017). Genus *Kitasatospora*, taxonomic features and diversity of secondary metabolites. J Antibiot.

[CR46] Tamura K, Stecher G, Kumar S (2021). MEGA11: molecular evolutionary genetics analysis Version 11. Mol Biol Evol.

[CR47] Tanvir R, Sheikh AA, Javeed A, Atta Ur R (2019). Chapter 11 - Endophytic Actinomycetes in the Biosynthesis of Bioactive Metabolites: Chemical Diversity and the Role of Medicinal Plants. Studies in Natural Products Chemistry.

[CR48] Tatchell RJ, Araman SF, Boctor FN (1972). Biochemical and physiological studies of certain ticks (ixodoidea). Z Parasitenkd.

[CR49] Thompson JD, Gibson TJ, Plewniak F, Jeanmougin F, Higgins DG (1997). The CLUSTAL_X windows interface: flexible strategies for multiple sequence alignment aided by quality analysis tools. Nucleic Acids Res.

[CR50] Toghueo RM, Boyom FF (2019). Endophytes from ethno-pharmacological plants: sources of novel antioxidants—a systematic review. Biocatal Agric Biotechnol.

[CR51] Trott O, Olson AJ (2010). AutoDock Vina: improving the speed and accuracy of docking with a new scoring function, efficient optimization, and multithreading. J Comput Chem.

[CR52] Trujillo ME, Alonso-Vega P, Rodríguez R, Carro L, Cerda E, Alonso P, Martínez-Molina E (2010). The genus *Micromonospora* is widespread in legume root nodules: the example of *Lupinus angustifolius*. ISME J.

[CR53] Usuki H, Nitoda T, Ichikawa M, Yamaji N, Iwashita T, Komura H, Kanzaki H (2008). TMG-chitotriomycin, an enzyme inhibitor specific for insect and fungal β-N-Acetylglucosaminidases, produced by actinomycete *Streptomyces anulatus* NBRC 13369. J Am Chem Soc.

[CR54] White JF, Kingsley KL, Zhang Q, Verma R, Obi N, Dvinskikh S, Elmore MT, Verma SK, Gond SK, Kowalski KP (2019). Review: Endophytic microbes and their potential applications in crop management. Pest Manag Sci.

[CR55] Yadav R (2013). Guidelines for laboratory and field testing of long-lasting insecticidal nets.

[CR56] Zhang Z, Schwartz S, Wagner L, Miller W (2000). A greedy algorithm for aligning DNA sequences. J Comput Biol.

[CR57] Zhao H, Yang A, Zhang N, Li S, Yuan T, Ding N, Zhang S, Bao S, Wang C, Zhang Y, Wang X, Hu L (2020). Insecticidal Endostemonines A-J Produced by endophytic *Streptomyces* from *Stemona sessilifolia*. J Agric Food Chem.

