# Supplementary material for: Endophytic actinobacteria from wild medicinal plants are a natural source of insecticide to control the African cotton leafworm (Spodoptera littoralis)
Source: AMB Express. 2023 May 15;13:47. doi: 10.1186/s13568-023-01550-x (PMC10185727; doi:10.1186/s13568-023-01550-x)
Supplement: Supplementary file 1 — Additional file 1: Figure S1. Phylogenetic tree of the strain Streptomyces sp. ES2 and the most related type strains, based on partial 16S rRNA gene sequences. Figure S2. Positive mode – Base Peaks Compounds (BPC) of ethyl acetate extract for Streptomyces sp. ES2 crude metabolite using LC-QTOF-MSMS. Chromatogram was shown as intensity relative retention time. Figure S3. Highlighted pharmacophoric regions (aromatic, polar, and nonpolar moieties) for the compounds with reported insecticide activities. Table S3. Lethal effects of actinobacteria crude extracts on the fourth instar larvae of laboratory Spodoptera littoralis (L-larvae). [file 13568_2023_1550_MOESM1_ESM.docx]

**Additional Material**

Journal: **AMB Express**

TITLE: Endophytic actinobacteria **from wild medicinal plants** are a natural source of insecticide to control the African cotton leafworm (*Spodoptera littoralis*)

Authors: **Mohamed** **K.** **Diab^1^. Hala M. Mead^1^. Mohamad A. Khedr^2^. Mohamed S. Nafie^3^.** **Abdelghafar M. Abu-Elsaoud^4^. Amro Hanora^5^. Sahar A. El-Shatoury* ^4^**

*^1^Agricultural Research Center, Plant Protection Research Institute, Pest Physiology Department, 12311 Giza, Egypt*

*^2^Agricultural Research Center, Plant Protection Research Institute, Cotton Leafworm Department, 12311 Giza, Egypt*

*^3^Suez Canal University, Faculty of Science, Chemistry Department, 41522 Ismailia, Egypt*

*^4^Suez Canal University, Faculty of Science, Botany & Microbiology Department, 41522 Ismailia, Egypt*

^5^*Suez Canal University, Faculty of Pharmacy, Microbiology Department, 41522 Ismailia, Egypt*

Corresponding author

**El-Shatoury S:** [sahar_hassan@science.suez.edu.eg](mailto:sahar_hassan@science.suez.edu.eg); orcid.org/0000-0002-6093-5145; telephone, +201222520880

*****

**Figure S1.** **Phylogenetic tree of the strain *Streptomyces* sp. ES2 and the most related type strains, based on partial 16S rRNA gene sequences.**

The evolutionary history was inferred by using the Maximum Likelihood method and Tamura 3-parameter model (Tamura 1992).The bootstrap consensus tree inferred from 1000 replicates (Felsenstein 1985) is taken to represent the evolutionary history of the taxa analyzed. Branches corresponding to partitions reproduced in less than 50% bootstrap replicates are collapsed. The percentage of replicate trees in which the associated taxa clustered together in the bootstrap test 1000 replicates) are shown next to the branches (Felsenstein 1985). Initial tree(s) for the heuristic search were obtained automatically by applying Neighbor-Join and BioNJ algorithms to a matrix of pairwise distances estimated using the Tamura 3 parameter model, and then selecting the topology with superior log likelihood value. This analysis involved 29 nucleotide sequences. All positions containing gaps and missing data were eliminated (complete deletion option). There were a total of 1268 positions in the final dataset. Evolutionary analyses were conducted in MEGA11 (Tamura et al. 2021).


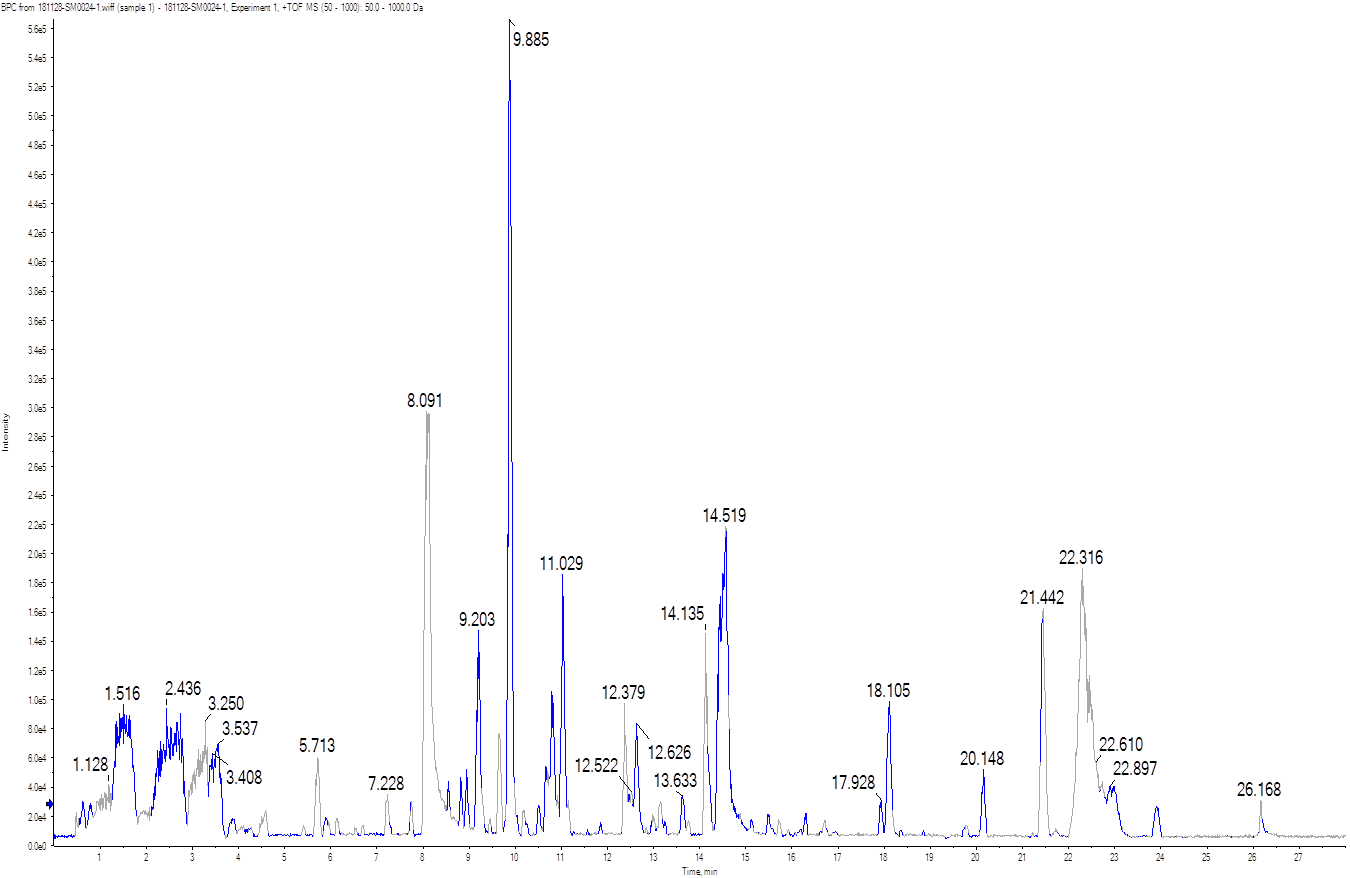


**Figure S2.** Positive mode – Base Peaks Compounds (BPC) of ethyl acetate extract for *Streptomyces* sp. ES2 crude metabolite using LC-QTOF-MSMS. Chromatogram was shown as intensity relative retention time.

**Figure S3.** Highlighted pharmacophoric regions (aromatic, polar, and nonpolar moieties) for the compounds with reported insecticide activities. Aromatic moiety with phenyl rings, polar moiety are nitro and phosphate groups, and non-polar moiety are alkyl chains of Carbon and Hydrogens.

**S1:** **Insect breeding protocol (Mansour et al. 1966)**

The laboratory adult cotton leafworms were fed 10% bee honey solution-soaked cotton wool plugs as a food source. The cotton plugs were renewed daily to avoid the fermentation or contamination. Fresh oleander leaves, *Nerium oleander* L., were provided in the adult jars as oviposition sites. After sexual mating between moths, the female moths lay most of their egg masses on the oleander leaves which were collected daily, transferred to new sterilized rearing jars and incubated to three-seven days until hatching.

After egg hatching, the newly pre-hatching larvae were transferred to new sterilized rearing jars. The larval instars were fed fresh castor bean leaves, *Ricinus communis* L., as a source of fresh food daily. Fresh castor leaves were collected daily from the Plant Protection Research Institute experimental field, washed and left to dry before putting to feed the larvae. The optimum number of castor bean leaves is (two: three) leaves of 25 × 25 cm per 40 larvae according to the larval instar phase. The rearing larval jars were incubated for 14 – 21 days passing through six larval instars till pupation stage. All breeding jars were covered with a muslin cloth held with a rubber band. The feeding protocol establishes for the replacement of food of the day before with a new fresh food daily to maintain the laboratory insect strain. All rearing jars contained sawdust or three layers of filter paper to absorb any surplus moisture and provide pupation sites at larval stage completion.

Before the pupation stage, the insects were undergoing pre-pupae period, which has taken 24 – 36 hours, forming a cocoon of sawdust or filter paper simulating to nature. Upon pupation, the formed pupae were collected, placed in cleaned jars, and incubated for seven-eight days until the new adult moths’ output. All nurturing larval jars were cleaned daily during all stages of the insect life cycle to avoid any contamination. The cleaning protocol is done through daily food replacement with the removal and cleaning of the jars, due to the larvae’ secretions resulting from nutrition and molting to ensure moisture control.

*S.* *littoralis* has six larval instars; the instars from the first to the third are limited in its movement, depending on silk threads. The assessment was performed on the fourth instar larvae because at that stage the insect has the highest strength and ability for movement between plants, for an extended distance, causing significant losses. The experiments were conducted on both L- and F- larvae

**S2:** **Non-targeted metabolomic analysis**

The LC-QTOF-MSMS analysis was performed using a Triple TOF^®^ 5600+, Sciex (ISO9001), Canada; fused two LC columns, In-Line filter disks Pre column (0.5 µm × 3.0 mm; Phenomenex Co., USA) and XBridge C18 column (3.5 µm, 2.1 × 50 mm; Waters Co., USA) maintained at 40°C.

The mobile phases consisted of deionized-water contains 0.1% formic acid at positive mode (as mobile phase A), 5.0 mM ammonium formate buffer [pH=8] containing 1% methanol at negative mode (as mobile phase B), and 100% acetonitrile at positive / negative mode (mobile phase C) at a constant flow rate of 0.3 ml/min. A gradient condition was applied with the following program: the equilibration of the column at zero minute, (90% A or B–10% C); and then 1–21 min, (90% A or B-10% C); 21–25 min, (10% A or B–90% C); 25.01–28 min, (90% A or B–10% C) at a constant flow rate of 0.3 ml/min.

The sample was injected at the Positive TOF-MS mode. The run duration took 28 min. The number of cycles was 2584. The cycle time took 0.6502 seconds. The injection volume was 10 µl. The mass spectrometry was performed on a Triple TOF^®^ 5600+ system. The following optimized parameters were used: curtain gas, 25 psi; nebulizer gas (gas 1), 45 psi; heater gas (gas 2), 45 psi; ion source heater 500°C; ion spray voltage, 4.5 kV; declustering potential, 80 eV; collision energy, 35 eV; Collision energy spread, 20 eV.

**A. Sample preparation**

Prepare mobile phase working solution (MP-WS): [(DI-Water: Methanol: Acetonitrile) as (50: 25: 25, respectively). Add 1 ml of MP-WS to 50 mg weighted sample. Put the mixture on a Vortex for two minutes followed by ultra-sonication for 10 min. Centrifugation for five minutes at 10000 rpm. 20 µl of stock (50/ 1000 µl) was diluted with 1000 µl reconstitution solvent. Finally, the injected concentration was 1µg/µl.

**B. Acquisition method "Injection in the instrument"**

Inject 25 µl on positive modes. Then, inject 25 µl of MP-WS as a blank sample.

**C. Data processing**

MasterView was used for feature (peaks) extraction from Total ion chromatogram (TIC), using (PeakView 2.2 Software Sciex), based on the following criteria; features should have Signal-to-Noise greater than five (non-targeted analysis). Non-targeted peaks finding and clustering were analyzed by (MarkerView 1.3 software, Sciex). Features intensities of the sample-to-blank should be greater than five. MarkerView was used for features annotation and removing isotopic peaks. MasterView was used again to identify peaks based on their fragments using: Build-in database (Data acquisition Analyst TF 1.7.1 software, Sciex) and online databases.

**Table S3:** **Lethal effects of actinobacteria crude extracts on the fourth instar larvae of laboratory *Spodoptera littoralis* (L-larvae)**

| **Actinobacteria strain ^a^** | | **Mortality in laboratory *S. littoralis*** | | **Actinobacteria strain** | | **Mortality in laboratory *S. littoralis*** | |
| --- | --- | --- | --- | --- | --- | --- | --- |
| **Strain number** | **genus** | **Immediate^b^** | **Latent^c^** | **Strain number** | **genus** | **Immediate** | **Latent** |
| 1 | *Pseudonocardia* sp. | na | + | 37 | *Streptomyces* sp. | na | **+** |
| 2 | Unknown | na | na | 38 | *Streptomyces* sp. | na | na |
| 3 | *Nocardioides* sp. | na | na | 39 | *Nocardioides* sp. | na | **+++** |
| 4 | *Nocardiopsis* sp. | na | na | 40 | *Streptomyces* sp. | na | na |
| **5** | ***Nocardiopsis* sp.** | **na** | **+++** | 41 | *Streptomyces* sp. | na | na |
| 6 | *Nocardiopsis* sp. | na | na | 42 | *Streptomyces* sp. | na | na |
| 7 | *Nocardiopsis* sp. | na | + | 43 | *Streptomyces* sp. | na | na |
| 8 | *Nocardiopsis* sp. | na | na | 44 | *Streptomyces* sp. | na | na |
| 9 | *Nocardioides* sp. | na | na | 45 | *Nocardiopsis* sp. | na | na |
| 10 | *Nocardioides* sp. | na | na | 46 | *Streptomyces* sp. | na | na |
| **11** | ***Nocardioides* sp.** | **+++** | **+** | 47 | *Streptomyces* sp. | na | na |
| 12 | *Nocardioides* sp. | na | +++ | 48 | *Streptomyces* sp. | na | na |
| 13 | *Nocardioides* sp. | na | na | 49 | *Streptomyces* sp. | na | na |
| 14 | *Nocardioides* sp. | na | + | 50 | *Streptomyces* sp. | na | na |
| 15 | *Nocardioides* sp. | na | +++ | 51 | *Nocardioides* sp. | na | na |
| 16 | *Nocardioides* sp. | na | na | 52 | Unknown | na | na |
| 17 | *Nocardioides* sp. | na | na | **53** | *Streptomyces* **sp. ES2** | **+++** | **+** |
| 18 | *Nocardioides* sp. | na | + | 54 | *Streptomyces* sp. | na | na |
| **19** | ***Nocardioides* sp.** | **+++** | **+++** | 55 | *Streptomyces* sp. | na | na |
| 20 | *Nocardioides* sp. | na | ++ | 56 | *Kibdellosporangium* sp. | na | na |
| 21 | *Nocardioides* sp. | na | na | 57 | *Nocardioides* sp. | na | na |
| 22 | *Nocardioides* sp. | na | na | 58 | *Nocardia* sp. | na | na |
| 23 | *Nocardioides* sp. | na | na | 59 | *Streptomyces* sp. | na | **+** |
| 24 | *Streptomyces* sp. | na | na | 60 | *Streptomyces* sp. | na | na |
| 25 | *Streptomyces* sp. | na | ++ | 61 | *Streptomyces* sp. | na | na |
| **26** | ***Streptomyces* sp.** | **+++** | **+++** | **62** | ***Streptomyces* sp.** | **+++** | **+++** |
| 27 | *Streptomyces* sp. | na | **+** | 63 | *Streptomyces* sp. | na | **+** |
| 28 | *Streptomyces* sp. | na | **+** | 64 | *Streptomyces* sp. | na | na |
| 29 | *Streptomyces* sp. | na | na | 65 | Unknown | na | na |
| 30 | *Streptomyces* sp. | na | na | 66 | *Promicromonospora* sp. | na | na |
| 31 | *Streptomyces* sp. | na | na | **67** | ***Pseudonocardia* sp.** | **+++** | **+** |
| 32 | *Streptomyces* sp. | na | na | **68** | *Streptomyces* **sp.** | **+++** | **na** |
| 33 | *Streptomyces* sp. | na | **+++** | 69 | *Streptomyces* sp. | na | na |
| 34 | *Streptomyces* sp. | na | **+** | 70 | *Streptomyces* sp. | na | na |
| 35 | *Streptomyces* sp. | na | na | Control | | na | na |
| 36 | *Streptomyces* sp. | na | na | Solvent Control | | na | na |
|  |  |  |  | Radiant Control | | **+++** | na |

^a^ Source: the actinobacteria isolation and identification was previously published (El-Shatoury et al. 2013). Unknown: actinobacterial strain could not be identified by chemotaxonomy methods. Immediate toxicity of seven metabolites is highlighted in grey. ^b^, immediate death within 72 hrs; ^c^, late death of larval/pupal stages.

Control, normally fed *Spodoptera littoralis* larvae; Solvent Control, larvae received leaves treated with ethyl acetate solvent; Radiant Control, larvae received leaves treated with Radiant SC 12%; +++, 50-30 % mortality; ++, 29-11 % mortality; +, ≤ 10 % mortality of pupal stage; na, no activity). The experiment was performed in triplicates (each replicate consisted of four larvae).

**References**:

El-Shatoury SA, El-Kraly OA, Trujillo ME, El-Kazzaz WM, El-Din el SG, Dewedar A: (2013) Generic and functional diversity in endophytic actinomycetes from wild Compositae plant species at South Sinai - Egypt. Res Microbiol. 164:761-769. doi: 10.1016/j.resmic.2013.03.004

Felsenstein J (1985) Confidence limits on phylogenies: An approach using the bootstrap . Evol 39:783-791. doi: 10.1111/j.1558-5646.1985.tb00420.x

Mansour NA, Eldefrawi ME, Toppozada AR, Zeid M: (1966) Toxicological Studies on the Egyptian Cotton Leaf worm, *Prodenia* *litura*. VI. Potentiation and Antagonism of Organophosphorus and Carbamate Insecticides. J Econ Entomol. 59:307-311

Tamura K: (1992) Estimation of the number of nucleotide substitutions when there are strong transition-transversion and G+C-content biases. Mol Biol Evol. 9:678-687. doi: 10.1093/oxfordjournals.molbev.a040752

Tamura K, Stecher G, Kumar S: (2021) MEGA11: Molecular Evolutionary Genetics Analysis Version 11. Mol Biol Evol. 38:3022-3027. doi: 10.1093/molbev/msab120
